# Clinical experience with nanoparticle albumin-bound paclitaxel, a novel taxane anticancer agent, and management of adverse events in females with breast cancer

**DOI:** 10.3892/ol.2015.2954

**Published:** 2015-02-10

**Authors:** SEIKI TAKASHIMA, SACHIKO KIYOTO, MINA TAKAHASHI, FUMIKATA HARA, KENJIRO AOGI, SHOZO OHSUMI, RYOKO MUKAI, YORIKO FUJITA

**Affiliations:** 1 Department of Breast Oncology, National Hospital Organization Shikoku Cancer Center, Matsuyama, Ehime 791-0280, Japan; 2 Department of Nursing, National Hospital Organization Shikoku Cancer Center, Matsuyama, Ehime 791-0280, Japan; 3 Department of Pharmacy, National Hospital Organization Shikoku Cancer Center, Matsuyama, Ehime 791-0280, Japan

**Keywords:** nab-paclitaxel, breast cancer, management of adverse events

## Abstract

Nanoparticle albumin-bound paclitaxel (nab-paclitaxel) is currently approved in Japan for treatment of breast cancer. However, apart from phase I clinical trials, data regarding Japanese patients are scant. In the present study, the efficacy and safety of nab-paclitaxel therapy were retrospectively analyzed in 22 patients with advanced or metastatic breast cancer who were treated at the National Hospital Organization Shikoku Cancer Center between November 2010 and June 2012. The nab-paclitaxel was administered once every three weeks. The median age of the patients was 59 years. The tumors were estrogen-receptor positive and/or progesterone-receptor positive in 63.6% patients. None of the patients had HER2-positive breast cancer. The median number of treatment cycles was six (range, two to 12). Six patients exhibited a partial response; the response rate was 27.3% and the clinical benefit rate was 31.8%. The response rate and clinical benefit rate were higher in patients who received nab-paclitaxel as first- or second-line treatment. The median time to treatment failure was 127 days (range, 27–257). Major adverse events were peripheral neuropathy (59%; Grade 3, 9%), myalgia (59%), rash (45%), and nausea and vomiting (50%). The results suggest that nab-paclitaxel is a well-tolerated and clinically useful anticancer preparation.

## Introduction

Taxanes, along with anthracyclines, are a key component in chemotherapy for breast cancer. The Taxanes include paclitaxel and docetaxel. In Japan, weekly paclitaxel has been widely used due to good efficacy and high tolerability (1). However, since paclitaxel is relatively insoluble, polyoxyethylated castor oil (Cremophor^®^EL) and ethanol have served as solvents to enhance solubility. Consequently, patients must receive premedication with corticosteroids, antihistamines and histamine-2 receptor antagonists prior to administration of paclitaxel. Despite premedication, however, ~40% patients exhibit mild hypersensitivity reactions and ~3% have serious, life-threatening reactions (2). Premedication with polyoxyethylated castor oil may also result in peripheral neuropathy and alter the pharmacokinetics of paclitaxel (3). Paclitaxel also has other solvent-related problems: Only limited types of intravenous infusion sets may be used and treating patients who exhibit alcohol intolerance is difficult (4).

Nanoparticle albumin-bound (nab)-paclitaxel (Abraxane^®^) is a solvent-free, colloidal suspension of paclitaxel and human serum albumin. Compared with conventional preparations of paclitaxel, nab-paclitaxel has a number of advantages: i) No premedication to prevent hypersensitivity is required; ii) any type of intravenous infusion set may be used (with no requirement for in-line filters); iii) nab-paclitaxel may be used even in patients who are sensitive to alcohol; and iv) nab-paclitaxel may be administered at a higher dose over the course of a shorter time period than paclitaxel (5). A phase III controlled study comparing tri-weekly paclitaxel (175 mg/m^2^) with tri-weekly nab-paclitaxel (260 mg/m^2^) in female breast cancer patients, conducted outside of Japan, reported that nab-paclitaxel was significantly superior to paclitaxel in terms of response rate (33% versus 19%; P<0.001) and progression-free survival times (23.0 versus 16.9 weeks; hazard ratio=0.75; P=0.006) (5). Nab-paclitaxel has thus overcome the predominant disadvantages of paclitaxel, and exerts enhanced antitumor activity. The present study reports the clinical experience of female breast cancer patients treated with nab-paclitaxel, and describes the adverse event management. Written informed consent was obtained from all patients.

## Patients and methods

### Patients

Data regarding 22 women with advanced or recurrent breast cancer who received nab-paclitaxel in the National Hospital Organization Shikoku Cancer Center (Matsuyama, Japan) between November 2010 and June 2012 were retrospectively analyzed. The general condition of the patients who received nab-paclitaxel had to satisfy the following conditions: i) A histologically confirmed diagnosis of breast cancer; ii) an Eastern Cooperative Oncology Group performance status of 0 to 2; iii) adequate bone marrow function (white blood cell count, ≥4,000/μl; platelet count, ≥100×10^3^/μl); iv) adequate liver function (bilirubin levels, ≤1.5 mg/dl; aspartate aminotransferase and alanine aminotransferase levels, ≤2.5-fold the institutional upper limit of normal); v) adequate renal function (creatinine levels, ≤1.5 mg/dl); and vi) adequate cardiac function.

### Treatment

Nab-paclitaxel was administered as a continuous intravenous infusion over the course of 30 min every three weeks. The patients did not receive any particular premedication.

### Response and toxicity assessment

Computed tomography and magnetic resonance imaging scans were performed at baseline and after three months to assess the radiological response of each patient according to the Response Evaluation Criteria in Solid Tumors, version 1.1 (6). The clinical benefit ratio (CBR) was defined as the percentage of patients who had a complete response (CR), partial response (PR) or stable disease. Adverse events were evaluated according to the Common Terminology Criteria for Adverse Events, Japanese version 4.0 (Japan Clinical Oncology Group/Japan Society of Clinical Oncology edition) (7). Time to treatment failure (TTF) was defined as the time period between the initiation of treatment and the cessation of treatment for any reason, including progressive disease, treatment-related toxicity and fatality, and was estimated by the Kaplan-Meier method.

### Countermeasures against adverse events

In the National Hospital Organization Shikoku Cancer Center, pharmacists provide patients with a detailed explanation with regard to the time periods when greatest bone marrow suppression occurs (nadir white-cell count), the countermeasures against infection, and the management of fever prior to chemotherapy and prior to discharge, using a patient compliance manual. Subcutaneous injection of granulocyte colony-stimulating factor (G-CSF) and treatment with antibacterial agents requires consideration in patients with grade 3 or higher febrile neutropenia, or grade 4 neutropenia.

In patients with peripheral neuropathy, treatment withdrawal or dose reduction was performed as required, and symptomatic treatment with vitamins, Gosha-jinki-gan, pregabalin (Lyrica^®^) and/or analgesics, such as loxoprofen (Loxonin^®^), was administered. Prophylactic treatment for myalgia and arthralgia was not administered, but nonsteroidal anti-inflammatory drugs (Loxonin and Mohrus^®^) were prescribed as required. The patients were informed in advance with regard to when these symptoms were most likely to occur and were instructed to take the prescribed drugs, so as to avoid enduring pain.

In our center, patients who receive nab-paclitaxel monotherapy are not usually administered antiemetics. The initial dose of nab-paclitaxel is administered during hospitalization, and the second and subsequent doses are prescribed on an outpatient basis. Pharmacists provide patients with drug management counseling prior to treatment, including information regarding drug names, treatment goals, treatment schedules, and potential adverse events with possible times of onset and countermeasures. Pharmacists are stationed in outpatient clinics and interview patients with regard to adverse events.

Nurses at the center provide patients with guidance concerning daily activities, accounting for the background characteristics of each patient. The nurses also describe the typical patient experience (development and management of adverse events), thereby attempting to relieve anxiety. In addition, the nurses provide patients with information regarding the severity of adverse events that would require treatment withdrawal or dose reduction, or the possibility of switching to other regimens, and the patients may seek consultation at any time.

## Results

### Patients

The clinical characteristics of the patients are shown in Table I. The median age at the initiation of treatment was 59 years (range, 35 to 73). A total of 18 patients exhibited postoperative recurrence and four had stage IV disease. The hormone receptor (HR) and human epidermal growth factor receptor-2 (HER2) protein expression status of patients was as follows: HR-positive and HER2-negative in 14 patients, and HR-negative and HER2-negative in 8 patients. No patient had HER2-positive tumors. The metastatic sites were the lymph nodes in 15 patients, liver in 12, lung in 11, bone in seven, pleura in five and skin in one. A total of 15 patients had metastases to multiple organs. The starting dose of nab-paclitaxel was the standard recommended dose (260 mg/m^2^) (8) in 13 patients and a reduced dose (179 to 240 mg/m^2^) in nine patients. Nab-paclitaxel was administered as a first-line treatment for metastasis/recurrence in 10 patients, a second-line treatment in four, and a third-line or subsequent treatment in eight. Prior to the nab-paclitaxel treatment, five patients had received capecitabine, four received gemcitabine, one received doxorubicin (Adriamycin^®^) plus cyclophosphamide, one received eribulin, one received S-1, two received paclitaxel and three received docetaxel.

### Efficacy

**Figure 1:**
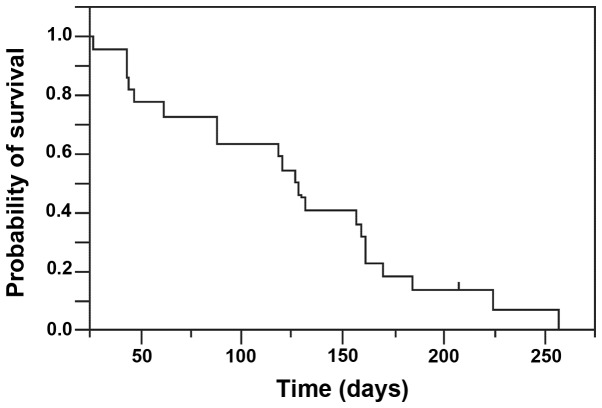
Time to treatment failure (TTF) in patients who received nab-paclitaxel. The median TTF was 127 days (range, 27–257).

Among the 22 patients, none had achieved a CR, but six reached a PR (response rate, 27.3%; 95% confidence interval, 8.7–45.9%). The CBR was 31.8% (Table II), and the median number of treatment courses was six (range, two to 12). The TTF was 127 days (range, 27 to 224), and treatment is being continued in one patient (Fig. 1). With regard to the association between response and HR expression status, the response rate did not differ between patients with HR-positive tumors and those with HR-negative tumors. The disease-free survival times did not differ between patients who responded to treatment and those who did not, or between patients who exhibited clinical benefits and those who did not. None of the five patients who had previously received taxanes responded to nab-paclitaxel. The response rate and CBR were markedly higher in patients with metastasis to a single organ than in those with metastases to multiple organs.

### Toxicity

The adverse events that developed during treatment with nab-paclitaxel are shown in Table III. Peripheral neuropathy occurred in 13 patients (59.1%), two of which exhibited grade 3 reactions. The mean number of treatment cycles at the onset of peripheral neuropathy was 2.5 (range, one to nine). Other adverse events reported included arthralgia and myalgia in 13 patients each, fever in four, rash in 10, nausea and vomiting in 11 patients each, diarrhea in four and stomatitis in four. These adverse events were mild and generally appeared only during the first course of treatment. The hematological toxicity symptoms (grade 3 or higher) reported included leukopenia and neutropenia in 12 patients (54.5%) each, but no febrile neutropenia was detected. Treatment was postponed in only one patient and G-CSF was used in only one patient. No hypersensitivity reactions to nab-paclitaxel occurred, despite the absence of premedication and shorter administration time than paclitaxel (5). No nail changes or rashes were observed.

### Reasons for discontinuation of treatment and subsequent chemotherapy administered

Treatment was discontinued due to progressive disease in 11 patients and adverse events in 10 patients, nine of whom experienced peripheral neuropathy. Adverse events were the primary reason for the withdrawal of first-line treatment, and progressive disease was the predominant reason for the cessation of second- and third-line, or subsequent treatment. Following the withdrawal of nab-paclitaxel treatment, four patients received capecitabine, three received vinorelbine, two received doxorubicin plus cyclophosphamide, two received eribulin, two received S-1 and one patient received paclitaxel. The CBR was 50% (2/4) in patients who received capecitabine and 50% (1/2) in those who were administered S-1. However, no clinical benefit was observed in patients on other treatment regimens.

## Discussion

Several large-scale phase III studies of paclitaxel administered every three weeks have shown that the response rate ranges between 11 and 29%, and that the median TTP ranges between 3.6 and 5.0 months (1,7,9–12). In the present study, six patients exhibited a PR to nab-paclitaxel with a response rate of 27.3%, a CBR of 31.8% and TTF of 127 days. A total of 80% patients on first-line treatment received the full dose of nab-paclitaxel, 50% of those on second-line treatment and 40% of those on third-line or later treatment. In the patients who were receiving first- or second-line treatment, the response rate was ~60%, indicating high antitumor effectiveness. Few patients responded nab-paclitaxel administered as third-line or later treatment. The proportion of patients who discontinued nab-paclitaxel due to adverse events was 60% when the drug was provided as first-line treatment, 0% for second-line treatment and 20% for third-line or later treatment. The finding that the CBR was only marginally higher than the response rate may be due to the high withdrawal rate of first-line treatment due to adverse events. Peripheral neuropathy was the adverse event that most frequently required the withdrawal of treatment. Improved methods of managing peripheral neuropathy must therefore be established.

As peripheral neuropathy exerts a particularly marked impact on quality of life, the management of this adverse effect is a key determinant of the success and completion rates of taxane treatment. Peripheral neuropathy is considered to involve the binding of taxanes to microtubules in peripheral nerve cells, promoting the aggregation of intracellular microtubules. This abnormal aggregation of microtubules in neuronal cells is considered to disturb sensory nerve function, resulting in neuropathy, but a number of factors remain unclear (13,14). Countermeasures against peripheral neuropathy include dose reduction, cessation of treatment and supportive medical therapy. One study evaluating low-dose nab-paclitaxel reported a response rate of 51.2% and a progression-free survival time of 22.4 weeks in patients who received 175 mg/m^2^ of nab-paclitaxel every three weeks (15). Furthermore, the low-dose regimen was not associated with grade 3 or higher peripheral neuropathy. A number of studies have assessed the effectiveness of various drugs as prophylactic or supportive therapy for peripheral neuropathy. Several randomized, phase II studies have reported that amifostine reduces the incidence of grade 2 peripheral neuropathy, whereas other clinical trials have demonstrated no apparent effect of this drug on the incidence of peripheral neuropathy (16–21). Although glutamine (22), vitamins (23) and other agents have been shown to be somewhat effective, treatment remains to be established and the management of peripheral neuropathy is problematic. At present, the early detection of peripheral neuropathy and dose reduction or treatment withdrawal prior to the onset of severe symptoms remains essential. In the National Hospital Organization Shikoku Cancer Center, peripheral neuropathy is symptomatically treated with vitamins or Gosha-Jinki-Gan. Since detecting symptoms of peripheral neuropathy is difficult for healthcare providers, pharmacists and nurses working in the outpatient chemotherapy clinic are encouraged to actively ask patients regarding their condition to facilitate the early detection and effective management of adverse events before they become serious.

In Japan, breast cancer is the most common type of cancer among females. The peak incidence of breast cancer occurs in females in their late 40s to early 60s, which is somewhat earlier than that of other types of cancer (24). As numerous chemotherapeutic regimens for breast cancer may be administered on an outpatient basis, the majority of patients want to receive treatment while continuing life as usual. The management of adverse events during home care is thus important. Since breast cancer therapy produces a number of adverse effects that directly influence quality of life, such as nausea and vomiting, peripheral neuropathy and hair loss, it is important to reduce anxiety whenever possible by providing appropriate advice with regard to the management of these events, including the expected times of peak occurrence. Close communication with patients is also essential to the early detection of these adverse events, further increasing the importance of patient interviews with pharmacists and nurses. Information obtained by physicians, pharmacists, nurses and other medical professionals should be shared to ensure that therapy is delivered more effectively and safely.

Nab-paclitaxel may be used safely; our clinical experience suggests that nab-paclitaxel is most likely to be effective when provided as a second-line treatment for patients with metastasis to a single organ. Even if the effectiveness of nab-paclitaxel is only equivalent to that of other taxanes, this drug offers numerous advantages for patients as well as medical professionals, such as a shorter treatment time.

However, use of the standard recommended dose of nab-paclitaxel for first-line therapy was associated with a high rate of treatment withdrawal, due to peripheral neuropathy. Therefore, the management of peripheral neuropathy remains important. Countermeasures against peripheral neuropathy, including the development of novel drugs, are required. Although experience remains limited, the use of a lower dose of nab-paclitaxel has been reported to be associated with a lower risk of peripheral neuropathy, without compromising effectiveness (14). Maintaining a good balance between treatment effectiveness and the quality of life of patients who receive nab-paclitaxel is therefore a main aim of patient care; the use of a lower dose should be considered whenever feasible.

## Figures and Tables

**Table I:** Demographic characteristics of females with advanced breast cancer who received nab-paclitaxel between November 2010 and June 2012.

Clinical parameter	No. of patients
Age, years^a^	59.0 (35–73)
PS	
0	12
1	8
2	2
HR, HER2 status	
HR(+)/HER2(−)	14
HR(−)/HER2(−)	8
Metastasis	
(+)/(−)	22/0
Liver	12
Lung	11
Lymph nodes	10
Bone	7
Pleura	5
Skin	1
Number of metastatic sites	
1	7
2	4
≥3	11
Therapy	
1st line	10
2nd line	4
≥3rd line	8

a Median (range).

PS, performance status; HR, hormone receptor; HER2, human epidermal growth factor receptor-2.

**Table II:** Antitumor effectiveness of nab-paclitaxel in females with advanced breast cancer.

Response	No. of patients
Complete response	0
Partial response	6
Stable disease	1
Progressive disease	11
Not evaluable	4
Response rate (%)	27.3
Clinical benefit rate (%)	31.8

**Table III:** Adverse events following nab-paclitaxel treatment in female patients with advanced breast cancer.

	No. patients				Percentage of patients	
		
	Gr1	Gr2	Gr3	Gr4	All grades	≥Gr3
Leukopenia	4	5	8	4	95.4	54.5
Neutropenia	3	4	6	6	86.3	54.5
Thrombocytopenia	6	1	0	1	36.3	4.5
Anemia	8	5	3	0	72.7	13.6
AST	9	4	0	0	59.1	0.0
ALT	16	0	0	0	72.7	0.0
Creatinine	4	2	0	0	27.2	0.0
Peripheral neuropathy	5	6	2	0	59.1	9.1
Myalgia	12	1	0	-	59.1	0.0
Arthralgia	7	6	0	-	59.1	0.0
Malaise	11	0	-	-	50.0	-
Alopecia	0	22	-	-	100.0	-
Nausea	9	2	0	-	50.0	0.0
Vomiting	9	2	0	0	50.0	0.0
Diarrhea	3	1	0	0	18.2	0.0

Gr, grade; AST, aspartate aminotransferase; ALT, alanine aminotransferase.
